# Drug-drug interaction between diltiazem and tacrolimus in relation to CYP3A5 genotype status in Chinese pediatric patients with nephrotic range proteinuria: a retrospective study

**DOI:** 10.3389/fphar.2024.1463595

**Published:** 2024-09-03

**Authors:** Qiaoling Yang, Yan Wang, Xuebin Wang, Ping Wang, Boyu Tan, Yijun Li, Huajun Sun, Wenyan Huang, Hongxia Liu

**Affiliations:** ^1^ Department of Pharmacy, Shanghai Children’s Hospital, School of Medicine, Shanghai Jiao Tong University, Shanghai, China; ^2^ Institute of Chinese Materia Medica, Shanghai University of Traditional Chinese Medicine, Shanghai, China; ^3^ Department of Pharmacy, Clinical Medical College, Affiliated Hospital of Chengdu University, Chengdu, China; ^4^ Department of Nephrology and Rheumatology, Shanghai Children’s Hospital, School of Medicine, Shanghai Jiao Tong University, Shanghai, China

**Keywords:** tacrolimus, diltiazem, drug-drug interaction, CYP3A5, pediatric patients, nephrotic range proteinuria

## Abstract

**Background:**

Tacrolimus is widely used to treat pediatric nephrotic range proteinuria (NRP). Diltiazem, a CYP3A4/5 inhibitor, is often administered with tacrolimus, affecting its pharmacokinetic profile. The impact of this combination on tacrolimus exposure, particularly in CYP3A5*3 genetic polymorphism, remains unclear in pediatric NRP patients. This study aimed to evaluate the effects of diltiazem on tacrolimus pharmacokinetics, focusing on the CYP3A5*3 polymorphism.

**Methods:**

We conducted a retrospective clinical study involving pediatric NRP patients, divided into two groups: those receiving tacrolimus with diltiazem and those receiving tacrolimus alone. Propensity score matching (PSM) was used to balance the baseline characteristics between the groups. We compared daily dose-adjusted trough concentrations (C_0_/D) of tacrolimus in both the original and PSM cohorts. The influence of diltiazem on tacrolimus C_0_/D, stratified by CYP3A5*3 genetic polymorphism, was assessed in a self-controlled case series study.

**Results:**

Before PSM, the tacrolimus C_0_/D in patients taking diltiazem was significantly higher compared to those with tacrolimus alone (75.84 vs. 56.86 ng/mL per mg/kg, *P* = 0.034). This finding persisted after PSM (75.84 vs. 46.93 ng/mL per mg/kg, *P*= 0.028). In the self-controlled case study, tacrolimus C_0_/D elevated about twofold (75.84 vs. 34.76 ng/mL per mg/kg, *P* < 0.001) after diltiazem administration. CYP3A5 expressers (CYP3A5*1/*1 and *1/*3) and CYP3A5 non-expressers (CYP3A5*3/*3) experienced a 1.8-fold and 1.3-fold increase in tacrolimus C_0_/D when combined with diltiazem, respectively.

**Conclusion:**

Diltiazem significantly increased tacrolimus C_0_/D, with CYP3A5*3 expressers showing higher elevations than non-expressers among pediatric NRP patients. These findings highlight the importance of personalized tacrolimus therapy based on CYP3A5*3 genotypes in pediatric patients taking diltiazem.

## 1 Introduction

Nephrotic syndrome (NS) is clinically characterized by massive proteinuria, hypoalbuminemia, edema, and hyperlipidemia (Downie et al., 2017; Mattoo and Sanjad, 2022). Tacrolimus, a widely used immunosuppressive drug, is recommended by the Kidney Disease Improving Global Outcomes (KDIGO) guidelines for treating NS in children and adults (Radhakrishnan and Cattran, 2012). Previous studies have shown that tacrolimus can reduce proteinuria by inhibiting the MAPK signaling pathway (Shen et al., 2016). However, the narrow therapeutic window of tacrolimus coupled with the potential for related toxicities present significant challenges and limitations for its clinical use (Brunet et al., 2019). Therefore, close therapeutic drug monitoring (TDM) and individualized dosing strategies are essential to ensure safe and effective administration of tacrolimus in patients.

Tacrolimus is primarily metabolized by the CYP3A enzyme in humans, leading to a high propensity for drug-drug interactions, particularly when taking CYP3A4 and CYP3A5 inhibitors or inducers (Niwa et al., 2007). Diltiazem, a CYP3A4/5 inhibitor, is often used in combination with tacrolimus to elevate the tacrolimus concentration in kidney transplant recipients and refractory nephrotic syndrome (RNS) based on its repressive roles on the metabolic elimination of tacrolimus and its protective roles from nephrotoxicity (Becker et al., 1996; Kidney Disease: Improving Global Outcomes KDIGO Transplant Work Group, 2009; Shen et al., 2016; Larpparisuth et al., 2019; Sun et al., 2019; Susomboon et al., 2022) Diltiazem has been reported to influence tacrolimus concentration by sharing the same CYP3A4/5 metabolic pathway (Sun et al., 2018; Larpparisuth et al., 2019). Initial studies in kidney transplant recipients have explored this interaction. A retrospective analysis from Canada found that diltiazem had a minimal effect on tacrolimus exposure in 64 adult kidney transplant recipients (Kothari et al., 2004). In contrast, a nonrandomized seven-period stepwise pharmacokinetic study conducted in Australia demonstrated that diltiazem significantly increased tacrolimus exposure by 26%–177% in renal transplant recipients, suggesting a substantial impact (Jones and Morris, 2002). Similarly, a Malaysian study reported a significant increase in tacrolimus exposure from 50% to 100% in kidney transplant recipients aged 18 years and older (Choong et al., 2018). Although these studies had limitations, such as only including adult recipients and not considering the effects of the CYP3A4 and CYP3A5 polymorphisms, they highlight the variability of the pharmacokinetic interaction between tacrolimus and diltiazem between different populations. Studies on the diltiazem-tacrolimus interaction in pediatric NS, particularly in China, are limited. Sun et al. initially reported a 43%–126% increase in whole blood tacrolimus trough levels (C_0_) in 7 pediatric patients with NS without analyzing the influences of the CYP3A genetic polymorphism (Sun et al., 2019). Subsequent studies confirmed the effects of CYP3A5*3 and diltiazem on tacrolimus pharmacokinetics (Guo et al., 2020; Li et al., 2021; Wang et al., 2021). The combination of CYP3A5*3 polymorphisms, concomitant use of diltiazem, body weight, and age significantly influenced daily dose-adjusted tacrolimus C_0_ (C_0_/D) (Guo et al., 2020; Li et al., 2021). In addition to CYP3A5*3, polymorphisms in CYP3A4, ABCB1, and SLCO1B3 could be related to the moderate effect of diltiazem on tacrolimus sparing (Wang et al., 2021). Several studies have shown that other factors, such as drugs (e.g., voriconazole, omeprazole, wuzhi capsules, phenobarbital), dietary supplements, herbs, and food, can also lead to individual differences in tacrolimus metabolism, distribution, and responsiveness (Bosó et al., 2013; Imamura et al., 2016; Wang et al., 2021; Zhao et al., 2021; Miedziaszczyk et al., 2022; Zhang et al., 2024).

Previous studies have demonstrated that the co-administration of diltiazem and tacrolimus leads to a significant drug-drug interaction, resulting in elevated tacrolimus trough levels (C_0_) in adult renal transplant recipients. Tacrolimus C_0_ levels increased significantly in CYP3A5 expressers compared to CYP3A5 non-expressers (Jones and Morris, 2002; Kothari et al., 2004; Li et al., 2011; Choong et al., 2018) However, the extent of the interaction between diltiazem and tacrolimus, influenced by the CYP3A5*3 polymorphism, remains unclear and insufficiently explored in pediatric NS patients. Addressing this gap is crucial for advancing clinical practice. To this end, we conducted a retrospective single-center study to investigate the effect of diltiazem on tacrolimus exposure and the impact of the CYP3A5*3 polymorphism in pediatric patients with nephrotic range proteinuria (NRP). This study provides insights that may inform clinical decision-making before routine co-prescription of diltiazem and tacrolimus.

## 2 Methods

### 2.1 Study design and subjects

This study followed the Declaration of Helsinki and received approval from the Ethics Committee of Shanghai Children’s Hospital (approval number: 2024R029-E01). Given that the results of this retrospective study did not influence further therapeutic decision-making, the Ethics Committee determined that formal consent was not required. The study comprises a cohort study and a self-controlled case series study.

#### 2.1.1 Part 1: cohort study

A retrospective cohort study was conducted on patients with NRP (urinary protein excretion >50 mg/kg/day (The Subspecialty Group of Renal Diseases, the Society of Pediatrics, Chinese Medical Association, 2017) who received tacrolimus between 1 January 2015 and 1 January 2022 at Shanghai Children’s Hospital. Patients who had taken other drugs that could affect tacrolimus blood levels, such as ketoconazole, itraconazole, verapamil, erythromycin, clarithromycin, voriconazole, omeprazole, wuzhi capsules, or phenobarbital were excluded. The patients were divided into two groups: those receiving tacrolimus with diltiazem and those receiving only tacrolimus. Clinical information including age, sex, body weight, height, pathological pattern, tacrolimus dosage, diltiazem dosage, blood concentrations, serum creatinine (Scr), serum albumin (ALB), hematocrit (HCT), alanine aminotransferase (ALT), aspartate aminotransferase (AST), and evaluate glomerular filtration rate (eGFR) were collected from the hospital information system and follow-up records.

#### 2.1.2 Part 2: self-controlled case series study

This part of the study used a self-controlled case series design, where each participant served as their control, thus automatically controlling for all fixed confounding variables that could alter the true association between exposure and outcome (Whitaker, 2008). Patients diagnosed with NRP who received tacrolimus and diltiazem between January 2015 and January 2022 were included following the same criteria as the retrospective study. This study examined the changes in dose-adjusted trough concentrations (C_0_/D) of tacrolimus before and after diltiazem administration.

### 2.2 Drug therapy regimen and determination of tacrolimus concentrations

All children received a combination treatment with tacrolimus and glucocorticoids. Tacrolimus was administered orally twice daily, with dosages tailored to trough concentrations (C_0_) obtained through TDM. The C_0_ of tacrolimus was measured before the sixth or seventh dose after achieving a steady-state concentration, with target levels set at 5–10 ng/mL. For patients who did not reach the target C_0_, diltiazem was administered to increase the blood concentration of tacrolimus, with a subsequent measurement of C_0_ before the sixth or seventh dose of tacrolimus after the initiation of diltiazem. For the patient who did not receive diltiazem, the tacrolimus concentrations collected for this study were those measured for the first C_0_ in patients at Shanghai Children’s Hospital. For the patient who received diltiazem, the tacrolimus concentrations collected for this study were those measured for first tacrolimus C_0_ after the initiation of diltiazem at Shanghai Children’s Hospital. Glucocorticoids, either prednisone or methylprednisolone, were administered once daily in conjunction with tacrolimus. The initial dose of prednisone was set at 1 mg/kg per day, with subsequent dose reductions. The methylprednisolone dose was converted to the equivalent prednisone dose, with 4 mg of methylprednisolone equal to 5 mg of prednisone.

Blood samples were collected from all patients 30 min before the morning dose. Tacrolimus C_0_ was determined according to the previous study (Yu et al., 2022). Briefly, whole blood samples were individually added into 300 μL of an internal standard extracting solution, which contained 3.0 ng/mL of ASC, 50 mmol/L of ZnSO4, and a methanol/water mixture at 50% concentration. Each sample within a centrifuge tube was subjected to vortex mixing for a duration exceeding 30 s, followed by a thorough blending for 5 min using a Vortex mixer V3 (Essenscien). Subsequent to a 5-minute centrifugation at 14,000 rpm while maintained at 4 °C, a minimum of 100 μL of the supernatant was carefully pipetted into a sample vial in preparation for LC-MS/MS analysis. The weight-adjusted daily dose and the tacrolimus daily C_0_/D were calculated.

### 2.3 DNA extraction and genotyping

Total DNA was extracted from whole blood samples obtained from subjects using a TIANamp Blood DNA Kit (Tiangen Biotech, Beijing). Genotyping of the SNP CYP3A5*3 (*rs776746*) was carried out by direct sequencing, as previously described (Liu et al., 2019). The primers used for CYP3A5*3 were:

Forward primer: 5′ACT​GCC​CTT​GCA​GCA​TTT​A 3′

Reverse primer: 5′CCA​GGA​AGC​CAG​ACT​TTG​A 3′

### 2.4 Statistical analysis

Data analysis was conducted using SPSS software (version 24.0, IBM, Armonk, NY, United States) and GraphPad Prism (version 7.0, GraphPad Software, CA, United States). Continuous variables are presented as mean ± standard deviation (SD) for normally distributed data or median (interquartile range, IQR) for data that are not normally distributed. Categorical data are described by frequencies and proportions. Propensity score matching (PSM) was used to reduce confounding biases. To minimize confounding biases between the group receiving tacrolimus comedicated with diltiazem and the group receiving tacrolimus alone, PSM was conducted to balance baseline characteristics (age, sex, body mass index, tacrolimus dosage, genotype, ALB, HCT, ALT, AST, and eGFR) between the group receiving tacrolimus with diltiazem and the group receiving tacrolimus alone. This was achieved using a 1:1 nearest-neighbor matching algorithm with a caliper set at 0.02. Differences in tacrolimus C_0_/D and other changes between groups were analyzed using the Mann-Whitney U test. The comparison of tacrolimus blood exposure before and after diltiazem co-administration was performed using a Wilcoxon signed-rank sum test. Statistical significance was defined as *p* < 0.05.

## 3 Results

### 3.1 Patient characteristics

Between 1 January 2015 and 1 January 2022, a total of 232 pediatric patients who received tacrolimus were initially identified. Of these, 93 patients were excluded because of the low level of urinary protein excretion (lower than 50 mg/kg/day). The following patients were further excluded: patients who had taken other drugs that could affect tacrolimus blood levels, such as ketoconazole, itraconazole, verapamil, erythromycin, clarithromycin, voriconazole, omeprazole, wuzhi capsules, or phenobarbital were excluded. Finally, a total of 117 patients (76 males and 41 females) were included. Among them, 96 patients were treated with tacrolimus, while 21 were taking diltiazem at a median dose of 1.82 mg/kg/day (range 1.56–2.67 mg/kg/day). A summary of the baseline characteristics of the patients is presented in Table 1. The groups differed primarily in terms of tacrolimus dosage and AST. After PSM, all baseline characteristics were well-balanced between the two groups. Ultimately, 21 patients were enrolled in each group: one group received tacrolimus and diltiazem, and the other received only tacrolimus.

**TABLE 1:** Demographic characteristics of patients before and after propensity score matching.

Variables	Before PSM			After PSM		
	Tacrolimus alone (n = 96)	Tacrolimus with diltiazem (n = 21)	*p*	Tacrolimus alone (n = 21)	Tacrolimus with diltiazem (n = 21)	*p*
Age (year)	8.33 (5.85,12.81)	10.58 (5.42,12.17)	0.612	7.83 (6.5,12.5)	10.58 (5.42,12.17)	0.725
body weight (kg)	32.93 (21.7,46.66)	40.9 (24.4,50.1)	0.162	27.85 (22.3,40.35)	40.9 (24.4,50.1)	0.187
Height (cm)	132.5 (114,153.75)	150 (122,161)	0.156	126.5 (119,152)	150 (122,161)	0.247
BMI (kg/m^2^)	18.01 (16.12,20.62)	19.11 (16.49,21.68)	0.493	18.11 (15.54,19.26)	19.11 (16.49,21.68)	0.358
Male (%)	60 (62.5)	16 (76.2)	0.234	15 (71.42)	16 (76.2)	0.726
ALB (g/L)	32.74 (24.67,39.93)	34.66 (27.46,37.39)	0.642	33.6 (27.11,40.05)	34.66 (27.46,37.39)	0.792
ALT (U/L)	14.5 (10,19.75)	16 (10,19)	0.923	11 (10,17)	16 (10,19)	0.42
AST (U/L)	21 (15,26)	16 (15,20)	0.033	17 (15,20)	16 (15,20)	0.82
BUN (mmol/L)	4.6 (3.53,6.08)	4.5 (3.5,6.8)	0.873	3.7 (3,5.4)	4.5 (3.5,6.8)	0.339
Scr (umol/L)	36.5 (28,47)	41 (34,55)	0.133	35 (31,47)	41 (34,55)	0.213
eGFR (mL/min/1.73 m^2^)	186.41 (157.08,211.26)	180.11 (129.35,197.76)	0.176	175.76 (160.63,210.73)	180.11 (129.35,197.76)	0.365
HCT (%)	39.35 (36.23,42.88)	39.4 (33.9,42)	0.420	39.3 (36.2,41)	39.4 (33.9,42)	0.801
Tacrolimus dosage (mg/kg/day)	0.09 (0.08,0.11)	0.08 (0.06,0.09)	0.041	0.09 (0.08,0.10)	0.08 (0.06,0.09)	0.195

Data are presented as median (interquartile range), ^*^ *p* < 0.05 compared to another group.

### 3.2 Genotype frequencies

The frequencies of the CYP3A5 genotype in each study group are presented in Table 2. Genotype frequencies were expected to adhere to the Hardy-Weinberg equilibrium proportions. The observed frequencies did not deviate significantly from these expectations (*p* > 0.05), indicating no significant differences in genotype distribution between the group receiving tacrolimus with diltiazem and the group receiving tacrolimus alone (*p* > 0.05).

**TABLE 2:** Distribution of CYP3A5 genotype in the study population of each group. *1/*1 and *1/*3: CYP3A5 expressers; *3/*3: CYP3A5 nonexpressers

Variant	Genotype	Frequency%(n)			
		Before PSM		After PSM	
		The group only receiving tacrolimus (n = 96)	The group receiving tacrolimus with diltiazem (n = 21)	The group only receiving tacrolimus (n = 21)	The group receiving tacrolimus with diltiazem (n = 21)
CYP3A5	*1/*1 and *1/*3	57.3% (55)	76.2% (16)	81.0% (17)	76.2% (16)
	*3/*3	42.7% (41)	23.8% (5)	19.0% (4)	23.8% (5)

### 3.3 Diltiazem significantly increased tacrolimus C_0_/D

**FIGURE 1:**
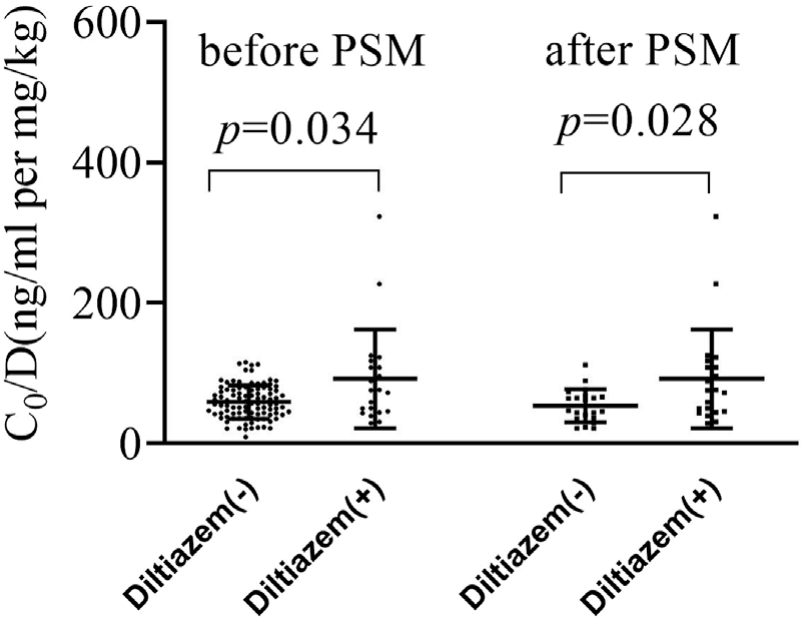
The change of tacrolimus C_0_/D by diltiazem in both the original and propensity score matching (PSM) cohort study. PSM, propensity score matching; C_0_/D, dose-adjusted trough concentrations; Diltiazem -: without diltiazem; Diltiazem +: with diltiazem; The horizontal lines represent the medium value and the interquartile range.

**FIGURE 2:**
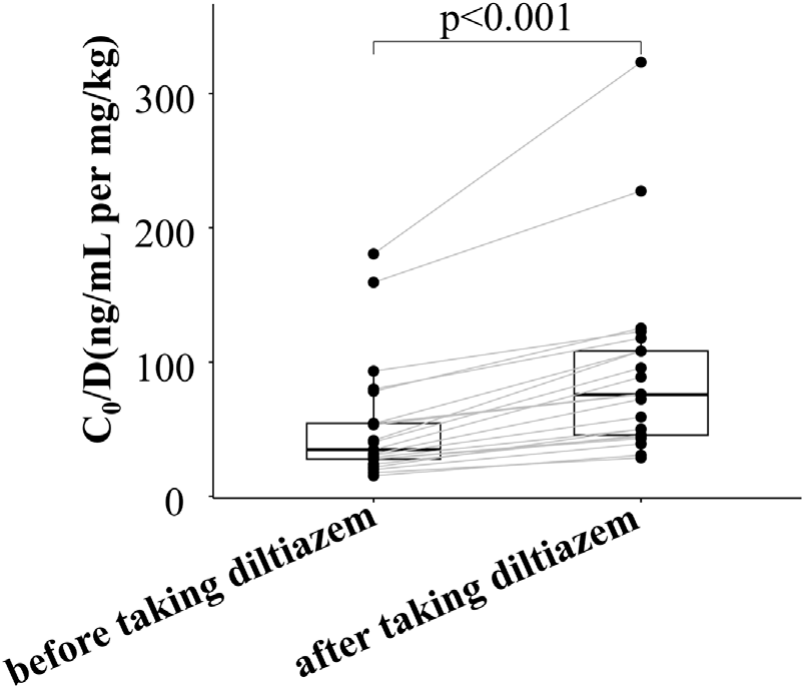
Change in tacrolimus C_0_/D of 21 patients before and after taking diltiazem in the self-controlled case series study. C_0_/D: tacrolimus dose-adjusted trough concentration; The horizontal lines represent the medium value and the interquartile range.

As shown in Figure 1 and Supplementary Table S1, the dose-adjusted C_0_ of tacrolimus in diltiazem patients was significantly higher than in those without diltiazem (75.84 vs. 56.86 ng/mL per mg/kg, *p* = 0.034). After matching, the post-PSM results were consistent with the initial findings, showing a significant difference (75.84 vs. 46.93 ng/mL per mg/kg, *p* = 0.028). In the 21 patients analyzed before and after adding diltiazem, tacrolimus C_0_/D increased nearly two-fold (75.84 vs. 34.76 ng/mL per mg/kg, *p* < 0.001), as shown in Figure 2 and Supplementary Table S2. These results demonstrate that diltiazem significantly increases tacrolimus C_0_/D.

### 3.4 Effect of the CYP3A5*3 genotype on tacrolimus C_0_/D and the extent of diltiazem–tacrolimus interaction in pediatric patients with NRP

**FIGURE 3:**
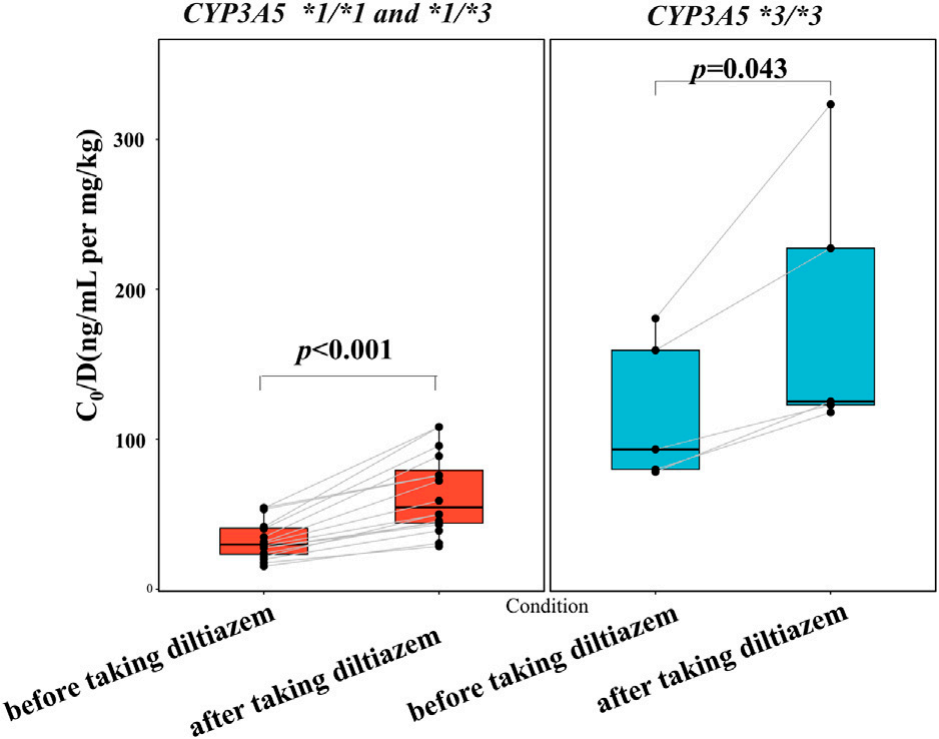
Impact of the CYP3A5 genotype on diltiazem-tacrolimus interaction in pediatric patients with NRP enrolled in a self-controlled case series study.

**FIGURE 4:**
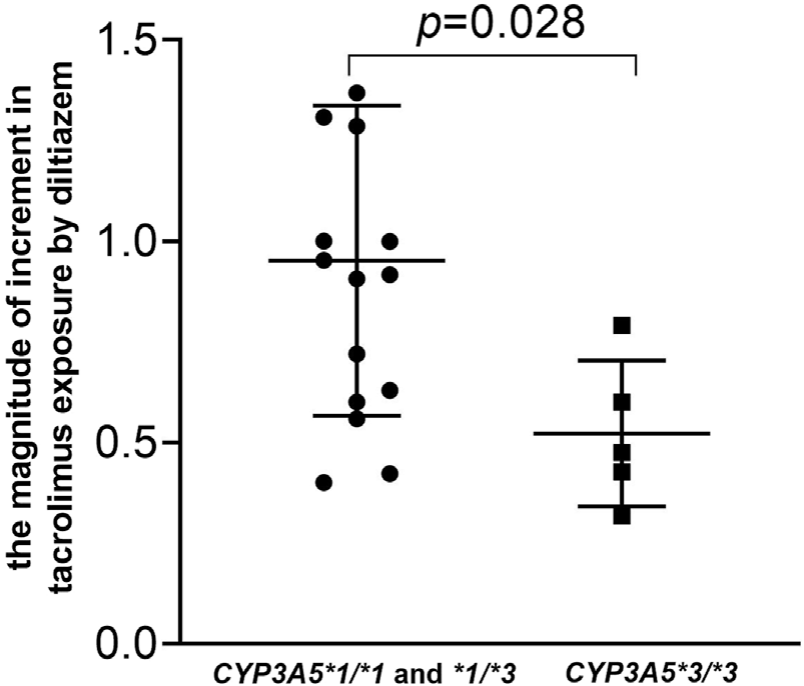
Impact of the CYP3A5 genotype on the magnitude of increase in tacrolimus by diltiazem in pediatric patients with NRP enrolled in a self-controlled case series study. The magnitude of increase were calculated using the following equation: The magnitude of increase = (Tacrolimus concentration of patients who received tiltiazem-Tacrolimus concentration of patients who did not receive diltiazem)/Tacrolimus concentration of patients who did not receive diltiazem.

As shown in Figure 3 and detailed in Supplementary Table S2, the current study highlights a significant drug-drug interaction between tacrolimus and diltiazem, particularly influenced by CYP3A5*3 polymorphisms. The results of our self-controlled case series study (n = 21) revealed that CYP3A5 expressers (CYP3A5*1/*1 and *1/*3) who received diltiazem exhibited a nearly 1.8-fold higher tacrolimus C_0_/D compared to CYP3A5 expressers who did not receive diltiazem (Figure 3, *p* < 0.001). Similarly, CYP3A5 nonexpressers (CYP3A5*3/*3) treated with diltiazem showed an approximately a 1.3-fold increase in tacrolimus C_0_/D compared to CYP3A5 nonexpressers without diltiazem (Figure 3, *p* = 0.043). A significant difference in the degree of increase in tacrolimus exposure due to diltiazem was observed between pediatric patients with different CYP3A5 genotype (Figure 4, *p* = 0.028), with CYP3A5 expressers experiencing a more pronounced increase in tacrolimus exposure when taking with diltiazem. These findings suggest that CYP3A5 expressers are more susceptible to the inhibitory effects of diltiazem on tacrolimus metabolism.

## 4 Discussion

The combination of tacrolimus with diltiazem, although recommended by the KDIGO clinical practice guidelines (Kidney Disease: Improving Global Outcomes KDIGO Transplant Work Group, 2009), is not commonly used in Chinese pediatric patients with NS. We explored the effect of diltiazem on tacrolimus C_0_/D in pediatric RNS patients and examined its relationship with CYP3A5*3 SNPs. Our findings indicate a significant increase in tacrolimus C_0_/D in pediatric patients when taking diltiazem, particularly among CYP3A5 expressers who showed significantly higher increases in tacrolimus exposure. The results offer valuable information for clinical management and will inform future prospective clinical trials in this patient group, potentially improving treatment outcomes and quality of life.

Previous studies have demomstrated the impact of diltiazem on systemic tacrolimus exposure (Jones and Morris, 2002; Choong et al., 2018; Larpparisuth et al., 2019; Lodha et al., 2020). Our observations are consistent with these reports, showing a significant increase in the tacrolimus C_0_/D ratio, irrespective of the baseline characteristics being balanced across the study population. We conducted an additional assessment through a self-controlled case series study to validate our findings. The consistency between this additional analysis and our primary results further corroborates the drug-drug interaction between diltiazem and tacrolimus, enhancing confidence in the validity and generalizability of our findings. Although the observed trend of increased tacrolimus exposure induced by diltiazem mirrors prior study, the variability in the magnitude of this increase underscores the complexity of predicting drug interactions. The magnitude of the increase in tacrolimus exposure caused by diltiazem varies and may be influenced by several factors. First, racial ancestry plays a significant role. Previous research has shown that the increase in tacrolimus exposure due to diltiazem ranged from 60% to 100% in Malaysian patients (Choong et al., 2018), compared to 26%–177% in Australian patients (Jones and Morris, 2002). These differences could be attributed to variations in genetic makeup or other factors that affect the extent of the diltiazem-tacrolimus interaction between Malaysian populations and Australian populations. Second, the dosage of diltiazem also impacts tacrolimus C_0_/D. Previous studies noted an increase in tacrolimus exposure in pediatric patients receiving diltiazem at 2.1 mg/kg/day (range 1.2–3.4 mg/kg/day) (Sun et al., 2019). Our study used a diltiazem dosage of 1.82 mg/kg/day (range 1.56–2.67 mg/kg/day), which could lead to varying effects on the tacrolimus C_0_/D. This suggests that the diltiazem dosage is critical in determining its influence on tacrolimus pharmacokinetics. To fully understand the impact of diltiazem dosage on tacrolimus exposure, further research involving various dosing regimens is necessary. Conducting extensive and well-designed studies across larger and more varied patient cohorts would provide deeper insight into the dynamics of this drug-drug interaction.

Previous studies have demonstrated that diltiazem competes with tacrolimus for the CYP3A5 binding site, reducing tacrolimus metabolism and increasing its exposure (Kuehl et al., 2001; Dai et al., 2006; Uesugi et al., 2006; Wang et al., 2021). This interaction is significantly affected by the CYP3A5*3 polymorphism. Higher sensitivity to the inhibitory effects of diltiazem on tacrolimus metabolism in CYP3A5 expressers was observed (Li et al., 2011). Similarly, our study observed that pediatric patients with CYP3A5 expressers exhibited significantly increased tacrolimus exposure when co-treated with diltiazem. These findings are consistent with previous research, confirming the crucial role of genetic variations in CYP3A5 in modulating the diltiazem-tacrolimus interaction (Kuehl et al., 2001; Dai et al., 2006; Uesugi et al., 2006; Li et al., 2011). The presence of the CYP3A5*1 allele, associated with increased enzymatic activity, leads to more potent CYP3A inhibition and increased tacrolimus C_0_/D. Wang et al. (2021) observed a 12.2% increase in tacrolimus C_0_/D in pediatric RNS patients taking diltiazem compared to those without (94.0 vs 83.8 ng/mL per mg/kg, *P* < 0.05), subjects with CYP3A5*1/*1 and *1/*3 experiencing a 54.5% increase compared to subjects with CYP3A5*3/*3. Our study found a significant increase in tacrolimus C_0_/D, with patients taking diltiazem experiencing a 61.6% increase. In the self-controlled case study, tacrolimus C_0_/D in CYP3A5 expressers increased 1.8-fold after combining with diltiazem, while CYP3A5 non-expressers increased 1.3-fold. These results are consistent with previous findings (Wang et al., 2021), highlighting the influence of CYP3A5 polymorphism on CYP3A enzyme activity, which is critical for pharmacotherapy decisions, particularly when managing drug interactions involving tacrolimus and diltiazem.

In addition, some clinical studies shown that diltiazem significantly affects tacrolimus pharmacokinetics by inhibiting CYP3A4 or P-glycoprotein (P-gp) (Jones and Morris, 2002; Guan et al., 2018). Among them, P-gp is encoded by ABCB1. CYP3A4*1G polymorphism and the ABCB1-C3435T polymorphism has been shown to affect tacrolimus exposure and the extent of its interaction with diltiazem in Chinese pediatric RNS patients (Wang et al., 2021). Research also indicates a linkage disequilibrium between CYP3A5 and CYP3A4 alleles in the Asian population, affecting drug interactions (Park et al., 2008). Interactions between CYP3A4 and CYP3A5 could be observed in Chinese kidney transplant recipients (Zuo et al., 2013). Diltiazem acts as a substrate for CYP3A4 and a potent inhibitor of CYP3A activity, could reduce tacrolimus (a CYP3A4/5 substrate) metabolism and increase tacrolimus exposure (Bosó et al., 2013; Imamura et al., 2016). The mechanism by which diltiazem affects blood exposure to tacrolimus may be attributed to its inhibition of CYP3A4. This inhibition induced by diltiazem leads to a decrease in overall CYP3A enzyme activity. As a result, the expression and metabolic activity of CYP3A5 are reduced, leading to increased exposure to tacrolimus. Patients carrying ABCB1-3435TT experienced greater increases in tacrolimus exposure due to reduced intestinal P-gp expression induced by ABCB1-3435TT mutation, which results in enhanced absorption (Wang et al., 2021). Interestingly, Diltiazem also competitively inhibits P-gp, enhancing the absorption of tacrolimus (Sakaeda et al., 2003). However, our study did not assess the CYP3A4*1G and ABCB1-C3435T polymorphisms and their involvement remains unexplored in this retrospective analysis. More research is necessary to fully understand these genetic influences on the interaction between diltiazem and tacrolimus.

This study has several limitations. First, the findings may lack generalizability since the data were obtained retrospectively from a single center. Conducting a multi-country and multi-center clinical investigation would enhance the precision and validity of our results. Second, the retrospective nature of the data collection means that the duration of tacrolimus treatment and other confounding factors such as diet could have varied between patients, potentially introducing confounding factors and bias. Third, the small sample size may limit statistical power to detect differences among smaller subgroups within our study, particularly the limited number of CYP3A5 non-expressers in the tacrolimus combined with the diltiazem group. A larger sample size would increase the reliability and robustness of the analysis. Forth, the interaction between corticosteroids and tacrolimus was not investigated, which is a limitation of our research. We will explore and clarify it in the future study. Lastly, although this retrospective study provides valuable preliminary information, validating the findings with prospective studies is imperative. Future prospective randomized controlled trials are needed to confirm these results and strengthen the evidence base, thus contributing to a more comprehensive understanding of the diltiazem-tacrolimus interaction in pediatric patients.

## 5 Conclusion

Our study demonstrates that diltiazem significantly increases the dose-adjusted trough concentration (C_0_/D) of tacrolimus in both cohort and self-controlled case series studies. CYP3A5 expressers are more susceptible to the inhibitory effects of diltiazem on tacrolimus metabolism compared to CYP3A5 non-expressers. These results underscore the need for personalized therapy approaches that incorporate the detection of the CYP3A5 genotype with TDM.

## Data Availability

The original contributions presented in the study are included in the article/Supplementary Material, further inquiries can be directed to the corresponding authors.

## Abbreviations

AST, Aspartate aminotransferase; ALT, Alanine aminotransferase; AUC, Area under curve; ALB, Albumin; BMI, Body mass index; BUN, Blood urea nitrogen; ETDA, Ethylene diamine tetraacetic acid; HCT, Hematocrit; PSM, Propensity score matching; Scr, Serum creatinine; TDM, Therapeutic drug monitoring; SNPs, Single nucleotide polymorphisms.
