# Supplementary material for: Drug-drug interaction between diltiazem and tacrolimus in relation to CYP3A5 genotype status in Chinese pediatric patients with nephrotic range proteinuria: a retrospective study
Source: Front Pharmacol. 2024 Sep 3;15:1463595. doi: 10.3389/fphar.2024.1463595 (PMC11405193; doi:10.3389/fphar.2024.1463595)
Supplement: Supplementary file 1 [file DataSheet1.docx]

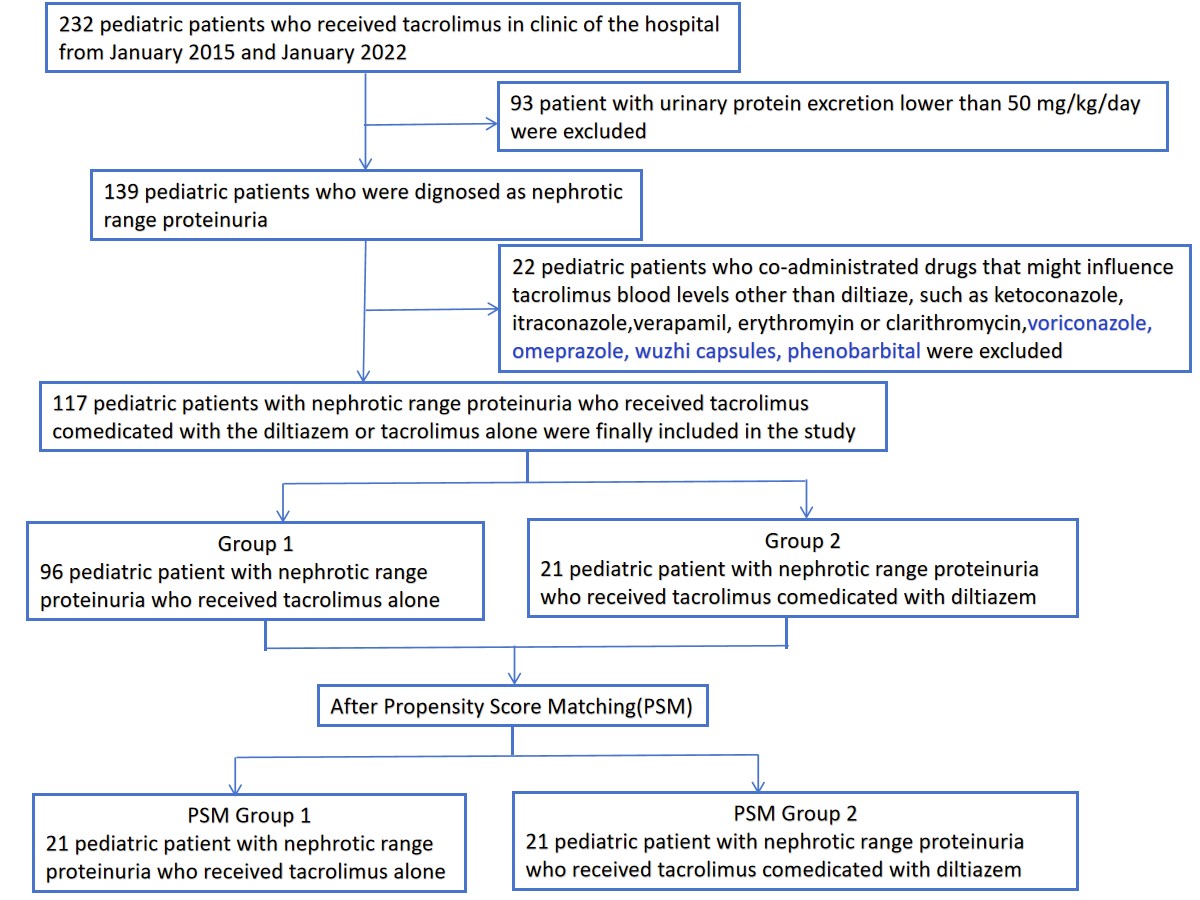


Supplementary Figure 1 the flow diagram of enrollment of patients

Supplementary Table 1. Diltiazem markedly increased the tacrolimus C_0_/D

| Variables | Before PSM | | After PSM | |
| --- | --- | --- | --- | --- |
|  | n | Tacrolimus C_0_/D (ng/ml/mg kg-1) | n | Tacrolimus C_0_/D (ng/ml/mg kg-1) |
| tacrolimus alone group | 96 | 56.86(41.7,76.63) | 21 | 46.93(36.01,66.25) |
| tacrolimus comedicated with diltiazem group | 21 | 75.84(45.55,108.24) | 21 | 75.84(45.55,108.24) |
| *p* |  | 0.034 |  | 0.028 |

Data are presented as median (interquartile range)

**Supplementary Table 2**. Impact of the CYP3A5 genotype on the diltiazem-tacrolimus interaction in pediatric patients when prescribed with diltiazem or without diltiazem.

| Variables | Tacrolimus C_0_/D (ng/ml/mg kg-1) | | *p* |
| --- | --- | --- | --- |
|  | prior to initiation of diltiazem treatment | after initiating diltiazem treatment |  |
| All (n=21) | 34.76.(27.65,54.39) | 75.84(45.55,108.24) | 0.00006 |
| CYP3A5 expressers (n=16) | 29.89(23.34,40.69) | 54.55(44.08,79.32) | 0.000438 |
| CYP3A5 nonexpressers  (n=5) | 93.28(79.91,159.38) | 125.25(122.85,275.42) | 0.043 |
| *P* | < 0.001 | < 0.001 |  |

Data are presented as median (interquartile range).
